# Dynamics of chemokines in severe drug hypersensitivity

**DOI:** 10.1186/2045-7022-4-S3-P29

**Published:** 2014-07-18

**Authors:** Hideo Asada, Kohei Ogawa, Ayako Hasegawa, Fumi Miyagawa, Hideaki Watanabe, Hirohiko Sueki, Mikiko Tohyama, Koji Hashimoto, Yoko Kano, Tetsuo Shiohara, Hiroyuki Fujita, Michiko Aihara

**Affiliations:** 1 Nara Medical University, Department of Dermatology, Japan; 2 Showa University School of Medicine, Department of Dermatology, Japan; 3 Ehime University Graduate School of Medicine, Department of Dermatology, Japan; 4 Kyorin University School of Medicine, Department of Dermatology, Japan; 5 Yokohama City University Graduate School of Medicine, Department of Environmental Immuno-Dermatology, Japan

## Background

Stevens-Johnson syndrome (SJS) / toxic epidermal necrolysis (TEN), and drug reaction with eosinophilia and systemic symptoms (DRESS) / drug-induced hypersensitivity syndrome (DIHS) are recognized as severe cutaneous adverse reactions (SCARs) usually induced by drugs. Due to the high risk of mortality, management of patients with SCARs requires rapid diagnosis. However, it is difficult to distinguish the early phase of SJS/ TEN and DIHS/DRESS from other ordinary types of drug-induced skin reactions. Therefore, there is a strong need of diagnostic markers for early stage of SCARs.

## Objective

In this study, we focused on chemokine productions in DIHS/DRESS and SJS/TEN. To determine whether chemokines might be useful markers for early diagnosis of SCARs, we examined the kinetics of chemokines during the course of the disease.

## Methods

Sera were obtained from 42 DRESS/DIHS patients associated with HHV-6 reactivation (n=31) and without HHV-6 reactivation (n=11), 16 SJS/TEN patients, and 17 patients with drug-induced maculopapular exanthema (MPE) in the acute and convalescent stage. Serum levels of Th1 chemokines (IP-10 and RANTES) and Th2 chemokines (TARC and MDC) were measured by ELISA.

## Results

We observed that serum TARC and MDC levels in patients with DRESS/DIHS were markedly higher than those in SJS/TEN and MPE patients in the acute stage (P<0.00003 in both cases). Furthermore, these levels are closely associated with HHV-6 reactivation. On the other hand, serum IP-10 showed significantly higher levels in SJS/TEN than DRESS/DIHS and MPE in the acute stage (P< 0.006 and P <0.0002, respectively), whereas there were no significant differences in serum RANTES levels among these three groups.

## Conclusion

We suggest that TARC and MDC could be useful diagnostic markers for DRESS/DIHS at early stage, and that IP-10, but not RANTES, might be a helpful indicator for differentiating SJS/TEN from other drug eruptions.

